# Leveraging transcranial ultrasound stimulation to enhance self-regulation in emotion and sleep

**DOI:** 10.3389/fnhum.2025.1594106

**Published:** 2025-12-19

**Authors:** Suraya Dunsford, Mica Komarnyckyj, Elsa Fouragnan

**Affiliations:** 1 School of Psychology, Faculty of Health, University of Plymouth, Plymouth, United Kingdom; 2 Brain Research and Imaging Centre, Faculty of Health, University of Plymouth, Plymouth, United Kingdom; 3 Manchester Biomedical Research Centre, Division of Psychology & Mental Health, University of Manchester, Manchester, United Kingdom

**Keywords:** self-regulation, emotion, sleep, transcranial ultrasound stimulation, neurofeedback, non-invasive brain stimulation, neurotechnology

## Abstract

This *Perspective* article discusses the emerging potential of transcranial ultrasound stimulation (TUS) as a non-invasive neuromodulatory technique for enhancing self-regulatory processes, particularly emotion and sleep regulation, in healthy individuals. Offering high spatial precision and the ability to target both cortical and deep brain regions, TUS uses focused ultrasound waves to induce acute and delayed effects on brain activity. We propose that combining TUS with neurofeedback methods and/or specific cognitive training exercises may capitalise on these neuroplastic effects, thereby augmenting and prolonging their impact to support lasting improvements in self-regulation. We focus on the domains of sleep and emotion regulation, where such an integrated approach may strengthen resilience and promote healthier functioning in the general population. Our aim is to highlight the potential of TUS-based integrated interventions for supporting mental health and well-being in non-clinical populations and to outline key directions for future research.

## 1 Introduction

Neuroscience is increasingly expected to become one of the defining fields of the modern world, with research advancements unlocking transformative potential on an unprecedented scale (Conroy, 2023; Nature, 2024). A key driver of this development is the rapidly growing field of neurotechnology, which encompasses a wide range of tools and techniques designed to interface with, measure, model, and perturb brain activity (Murphy and Fouragnan, 2024). Though some of these technologies are still in early or experimental stages, they hold significant promise for enhancing brain health, boosting cognitive function, and promoting overall mental well-being on a broad scale, provided that careful ethical considerations are addressed from the outset (Yuste, 2023).

Sleep and emotion regulation are fundamental processes that shape cognitive performance, resilience and overall well-being. When these processes function optimally within normative functional ranges they promote psychological stability and adaptability; when transiently dysregulated, they increase vulnerability to mental health difficulties (Aldao et al., 2010; Luff and de Lecea, 2024). Enhancing these regulatory processes is therefore central not only to clinical treatment but also to the prevention of illness and the promotion of mental well-being across the general population. Recent advancements in neurotechnology highlight a future where brain stimulation extends beyond treating disease, to enhance neurophysiological function and resilience at a general population level. With rising social stress and mental health burden (Baker and Kirk-Wade, 2024), developing effective methods to enhance self-regulation is increasingly urgent.

**Figure 1:**
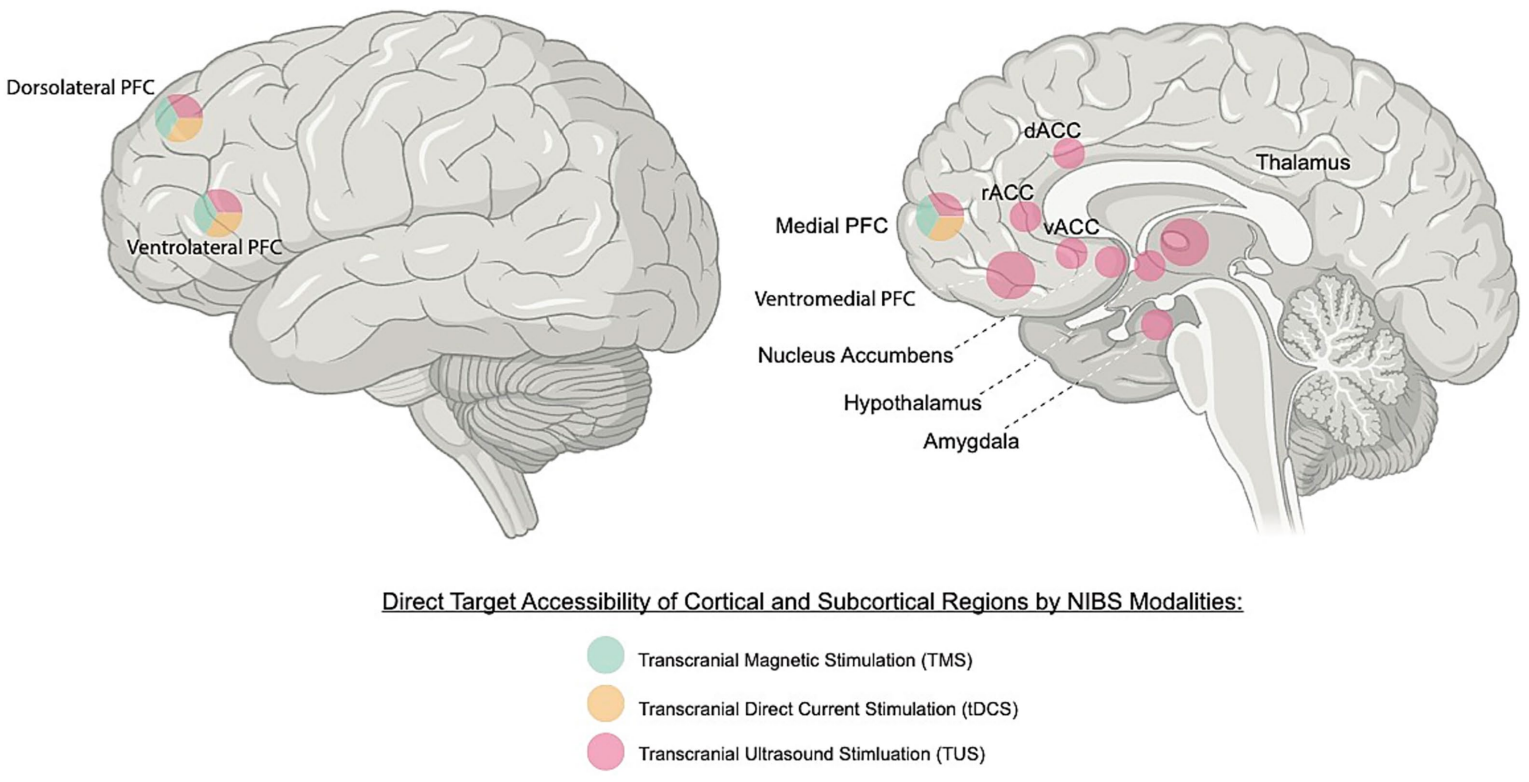
Potential non-invasive brain stimulation (NIBS) targets for emotion and sleep regulation. This figure illustrates cortical and subcortical regions involved in the regulation of emotion and sleep that represent potential targets for non-invasive brain stimulation. Lateral prefrontal areas, including the dorsolateral (DLPFC) and ventrolateral prefrontal cortex (VLPFC), as well as the medial prefrontal cortex (mPFC), are commonly targeted by transcranial magnetic stimulation (TMS) and transcranial direct current stimulation (tDCS) to modulate cognitive and affective control. Other deeper cortical regions, including the ventromedial prefrontal cortex (VMPFC) and anterior cingulate cortex (ACC), comprising of the dorsal (dACC), rostral (rACC), and ventral/subgenual (vACC/sgACC) subregions, and subcortical regions such as the nucleus accumbens (NAcc), amygdala, thalamus, and hypothalamus, lie beyond the direct reach of conventional TMS and tDCS. Transcranial ultrasound stimulation (TUS) offers a major advance by enabling access and specific targeting of both cortical and deep subcortical structures, providing a unique capability to directly modulate a wider network of emotion and sleep related regions that are inaccessible to traditional NIBS techniques.

Non-invasive brain stimulation (NIBS) has gained prominence through methods such as transcranial magnetic stimulation (TMS) and transcranial direct current stimulation (tDCS), which modulate cortical excitability (Figure 1) and induce plasticity (Polanía et al., 2018). Both techniques act through polarity or frequency dependent shifts in membrane potential, neurotransmitter balance and NMDA (N-methyl-D-aspartate) receptor-mediated plasticity, inducing long term potentiation (LTP) or depression (LTD) like effects, changes in cortical inhibition/excitation, and altered network connectivity largely confined to cortical regions (Yamada and Sumiyoshi, 2021; Jannati et al., 2023). They provide well established models of non-invasive neuromodulation, with clinical guidelines [National Institute for Health and Care Excellence (NICE), 2015b] now recommending repetitive TMS as an evidence-based treatment for depression (NICE IPG542). In contrast, tDCS remains an investigational research technique due to limited evidence on efficacy [National Institute for Health and Care Excellence (NICE), 2015a].

Building on this foundation, transcranial ultrasound stimulation (TUS) is emerging as one of the most promising neurotechnologies of the last decade. TUS delivers focused acoustic energy with millimetre precision, enabling modulation of both superficial and deep brain regions (Figure 1). It can induce immediate (“online”) effects as well as delayed longer-lasting (“offline”) changes supported by evoked neuroplasticity (Legon et al., 2018; Bault et al., 2024). With the ability to reach subcortical targets and selectively influence different cell types (Kubanek, 2018; Gaur et al., 2020; Murphy and Fouragnan, 2024; Murphy and de Lecea, 2024; Murphy et al., 2024), TUS offers unparalleled promise to advance neuroscientific research and personalised brain interventions in ways previously unexplored. Importantly, TUS operates within a very different parameter space from both diagnostic ultrasound imaging and ablative surgical ultrasound. Whereas imaging uses unfocused, higher-duty signals and ablation employs intensities sufficient to cause thermal coagulation, TUS applies low-intensity, pulsed and highly focused acoustic energy to modulate brain activity without damaging tissue.

**Figure 2:**
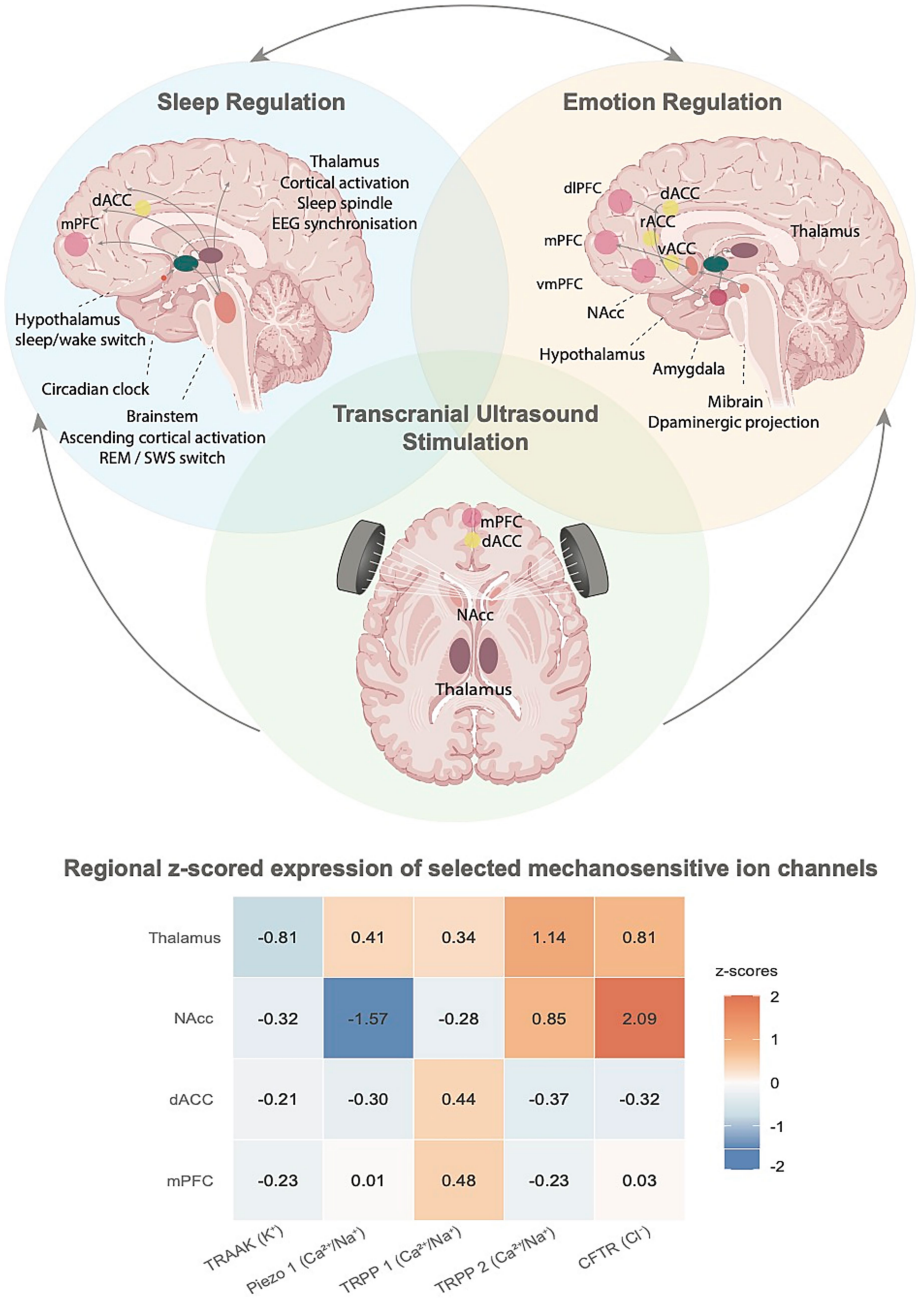
Overlapping brain networks involved in sleep and emotion regulation, and potential transcranial ultrasound stimulation (TUS) targets. The figure illustrates principal brain regions involved in sleep and emotion regulation. Arrows within the brain schematics indicate directionality within these networks. Bidirectional interactions exist between sleep and emotion, whereby alterations in one system can influence the other. Sleep: Key structures include the medial prefrontal cortex (mPFC), dorsal anterior cingulate cortex (dACC), thalamus, hypothalamus, and brainstem, which regulate processes such as circadian rhythms, sleep/wake switching, cortical activation, and EEG synchronisation. Emotion: Emotion regulation engages the dorsolateral prefrontal cortex (dlPFC), mPFC, ventromedial prefrontal cortex (vmPFC), anterior cingulate cortex (dACC, rACC, and vACC), amygdala, nucleus accumbens (NAcc), hypothalamus, and thalamus. The midbrain provides dopaminergic projections critical for motivation and emotional regulation. TUS may enhance self-regulation across both systems through modulation of shared regions such as the mPFC, dACC, NAcc, and thalamus. These effects are underpinned by the multi-level mechanisms of TUS described in the Introduction, ranging from ion channel modulation to network-level plasticity, which provide a mechanistic basis for its potential impact on both sleep and emotion regulation. The accompanying heatmap shows z-scored expression (Donor H0351.2002, Allen Human Brain Atlas) of mechanosensitive ion channel genes, selected based on evidence for mechanosensitive responses to ultrasound (TRAAK, Piezo1, TRPP1, TRPP2, CFTR), in key TUS target regions (mPFC, dACC, NAcc, thalamus; thalamic values from DTLd and DTLv). Expression levels: ≤ −1.2 very low, −1.2 to −0.4 low, −0.4 to +0.4 baseline, +0.4 to +1.2 high, ≥ +1.2 very high. Probes: A_32_P805871, A_23_P168916, A_23_P77502, A_23_P167324, A_23_P215720. These mechanosensitive channels represent a plausible molecular interface through which TUS could influence self-regulatory network dynamics underlying the regulation of both sleep and emotion.

TUS appears to influence brain activity through several converging mechanisms. At the cellular level, mechanical forces generated by the acoustic wave can deform the membrane and cytoskeleton, altering tension and thereby engaging mechanosensitive ion channels, such as TRPP1/2, Piezo1 and CFTR (see Figure 2; Yoo et al., 2022; Wen et al., 2023). These biophysical effects can shift the resting potential towards depolarisation or hyperpolarisation, producing parameter-dependent changes in action potential firing (Prieto et al., 2018; Prieto et al., 2020; Yoo et al., 2022; Clennell et al., 2023; Wen et al., 2023). Ultrasound may also interact with voltage-gated channels, for example, modulation of two-pore potassium channels, such as TRAAK, has been linked to hyperpolarisation and waveform changes, while effects on sodium channel kinetics may help explain the bidirectional influence on neuronal excitability observed in slice and animal studies (see Figure 2; Prieto et al., 2020; Prieto et al., 2018; Sorum et al., 2021).

Beyond single-cell physiology, evidence points to changes in neurotransmitter release and synaptic plasticity. In rodents, sonication of regions such as thalamic, hippocampal, striatal, and somatosensory have been associated with shifts in dopamine, serotonin, and GABA levels, as well as intensity-dependent induction of LTP or LTD like effects, mediated by NMDA and AMPA (*α*-amino-3-hydroxy-5-methyl-4-isoxazolepropionic acid) receptors, and brain-derived neurotrophic factor (Min et al., 2011; Yang et al., 2012; Huang et al., 2019; Niu et al., 2020; Niu et al., 2022; Qin et al., 2024).

Finally, TUS can influence large-scale networks. Studies in non-human primates and humans demonstrated altered functional connectivity within targeted circuits, with neuromodulatory effects lasting up to 2 h; however, further research is needed to establish the duration of these effects (Folloni et al., 2019; Fouragnan et al., 2019; Bongioanni et al., 2021; Yaakub et al., 2023b). Such findings suggest that ultrasound triggers a cascade of effects through multi-level interactions, ranging from changes in membrane capacitance and ion channel activity to alterations in whole-brain network dynamics. Through such mechanisms, TUS provides a potential means to normalise maladaptive patterns of neural activity and to enhance adaptive network dynamics, particularly when paired with training that directs plasticity towards functional processes. In the following sections, we discuss in detail how these mechanisms may be harnessed in self-regulation, namely the capacity to monitor and adapt one’s thoughts, emotions, and behaviours in pursuit of long-term goals (Carver and Scheier, 2011), in the domains of sleep and emotion.

TUS is already under active investigation in clinical trials (e.g., NCT06722339, NCT05147142, NCT04620460, and NCT05301036), where it has been proposed as an alternative to conventional pharmacological treatments for neurological and neuropsychiatric disorders (Hu et al., 2023; Mahoney et al., 2023; Shi and Wu, 2025). Early evidence suggests that TUS shows promise as an effective and well-tolerated intervention, while demonstrating a favourable safety profile (Legon et al., 2020; Yaakub et al., 2024; Murphy et al., 2025). Thermal and mechanical safety considerations are central. Stimulation protocols should conform to the human safety guidelines proposed by the ITRUSST consortium (Murphy et al., 2025), which ensure that temperature rise remains negligible and mechanical index values remain well below cavitation thresholds. In practice, computational simulations are routinely used before human application to optimise targeting, estimate in-situ pressures, confirm safety margins, and minimise transmission loss through the skull (Treeby and Cox, 2010; Miscouridou et al., 2022; Yaakub et al., 2023a). By targeting a specific brain region and influencing its functional circuit, rather than acting on the whole brain simultaneously, as pharmacological treatments typically do, TUS has the potential to reduce systemic side effects. The circuit-level selectivity helps avoid the widespread and often adverse reactions associated with drugs (Sarica et al., 2022).

Taken together, these safety and feasibility foundations open the possibility of preventative applications in healthy individuals. Importantly, we do not view TUS as a stand-alone preventative method, but as a priming approach to be paired with neurofeedback and/or targeted cognitive training, allowing stimulation-induced plasticity to be directed and consolidated through behaviour. This framework draws on evidence that NIBS can transiently shift neural excitability and plasticity thresholds, thereby facilitating the induction of longer-lasting, experience-dependent changes during therapy or practice (Saccenti et al., 2024). Accordingly, this *Perspective* explores how TUS may leverage such metaplastic mechanisms to improve self-regulation in healthy individuals, focusing on sleep and emotion regulation.

## 2 Sleep

Sleep is a reversible state of altered consciousness associated with physical inactivity and reduced responsiveness to stimuli. It is essential for maintaining both physical and mental health, consolidating memories and supporting the immune system. Poor sleep disrupts mood and cognitive functioning, and is often comorbid with psychiatric conditions (e.g., depression, anxiety, bipolar; Luff and de Lecea, 2024). Sleep is structured in alternating phases of rapid eye movement (REM) and non-rapid eye movement (NREM) sleep which have unique brain patterns and physiological states. REM is important for learning and memory and the deepest part of NREM, known as slow wave sleep, is considered the most restorative stage and therefore a target for many sleep improvement therapies (Dijk, 2009; Luff and de Lecea, 2024).

At the population level, poor sleep has become a major public health concern, driven by modern lifestyles, occupational stress, and the demands of a 24/7 economy (The Lancet Diabetes Endocrinology, 2024). Large-scale surveys report substantial current sleep difficulties, many chronic in nature (The Lancet Diabetes Endocrinology, 2024), with over one-third of US adults failing to obtain sufficient sleep on a regular basis [Centers for Disease Control and Prevention (CDC), 2024]. This growing prevalence underscores the urgency of developing effective strategies to improve sleep health and its downstream effects on emotional and cognitive well-being.

### 2.1 NIBS methods for sleep regulation

Several FDA approved drugs have been shown to improve sleep quality by enhancing slow wave sleep (e.g., Trazodone, Mirtazapine, Gabapentin, and Pregabalin; Walsh, 2009). Although these drugs can modestly increase slow wave activity and reduce sleep latency, they are associated with an unfavourable risk/benefit ratio characterised by tolerance, next-day sedation, dependence, and withdrawal upon discontinuation (Krystal et al., 2019). This highlights the broader need for safe, non-pharmacological approaches to support sleep quality across the general population.

NIBS techniques, such as TMS, tDCS and closed-loop auditory stimulation (CLAS), have been explored as ways to enhance restorative sleep and promote healthy cognitive and emotional functioning. Although TMS and CLAS can modulate cortical brain activity related to sleep and improve subjective sleep quality, there is little evidence these methods improve objective sleep measures (e.g., time in REM/NREM, total sleep time, time to fall asleep, number of wakeful episodes; Luff and de Lecea, 2024). Furthermore, TMS and tDCS do not have sufficient spatial specificity or stimulation depth to target subcortical structures (including thalamus, hypothalamus, and basal forebrain) which play a vital role in governing sleep (see Figure 1; Dijk, 2009; Crunelli et al., 2015; Falup-Pecurariu et al., 2021). These limitations may explain why, to date, no NIBS technique has achieved widespread clinical adoption for sleep improvement therapy (Luff and de Lecea, 2024).

### 2.2 TUS and sleep: existing research

Although TUS holds potential to address the limitations of tDCS and TMS, no studies investigating its effects on sleep in human have yet been published, although several are currently underway. Recent rodent studies show promising results, with offline TUS applied before sleep to the motor cortex and hippocampus shown to improve NREM sleep ratio and total sleep time (Wang et al., 2023). Online closed-loop systems, which monitor brain signal with EEG and apply TUS, during sleep demonstrate that TUS can increase REM sleep and protect spatial working memory when applied to the prefrontal cortex (Jo et al., 2022); and increase NREM ratio and decrease wakeful episodes when applied to the hippocampus (Dong et al., 2023). Furthermore, closed-loop TUS demonstrates the regulatory effect of TUS on different sleep states may depend on synchronisation of stimulation timing with specific hippocampal phases (slow oscillations and theta waves; Dong et al., 2023), highlighting this might be a key factor when translating findings into humans.

Outside of sleep literature, other notable TUS studies have focused on the thalamus, which is known to be a key regulator of slow-wave sleep (Crunelli et al., 2015), but have not yet delivered stimulation to this region within the objective of enhancing sleep quality (Grigoreva et al., 2025). These online and offline studies demonstrate the ability of TUS to safely and robustly activate this subcortical region (Legon et al., 2018; Murphy et al., 2022; Fan et al., 2024; Murphy and de Lecea, 2024), influencing resting state brain connectivity (Fan et al., 2024) and EEG theta and beta power (Legon et al., 2018).

### 2.3 Online TUS for sleep self-regulation

Aligning neuromodulation with the brain’s natural oscillatory phases during sleep has been recognised as a key component of the next generation of NIBS sleep improvement technologies (Luff and de Lecea, 2024). We therefore anticipate the next major research investment in this field will build on recent animal models (Jo et al., 2022; Dong et al., 2023) to evaluate human closed-loop TUS systems for sleep improvement. Recent patents illustrate this trend, describing a real-time human EEG monitoring system which detects sleep phases and optimises ultrasound stimulation focused on the thalamus, with the aim of enhancing slow waves to improve sleep (Mascaro et al., 2023; Murphy et al., 2023).

This approach’s feasibility in humans may be impacted by discomfort caused by wearing the device and noise generated by ultrasound stimulation (Kop et al., 2024). These challenges are particularly relevant for individuals experiencing sleep difficulties, who often show heightened sensitivity to external stimuli and sensations (Pieroni et al., 2024). However, although these are current limitations, future advancements in technology may mitigate these issues, particularly by reducing discomfort and optimising stimulation parameters to minimise noise, for example through waveform smoothing (Kop et al., 2025), thereby making this approach more viable in the future.

### 2.4 Offline TUS for sleep self-regulation

TUS-mediated neuroplasticity in subcortical regions lasts at least 50 min following stimulation (Yaakub et al., 2023b), therefore offline methods applying TUS prior to sleep could overcome discomfort issues associated with online TUS. Although many studies have shown that offline TUS is effective at inducing short-term neuroplasticity which spans a few hours post sonication (Folloni et al., 2019; Fouragnan et al., 2019; Clennell et al., 2021); the precise temporal dynamics and duration of these effects remain to be fully elucidated. Very few studies have capitalised on this neuroplastic state, which could be leveraged alongside relaxation techniques such as progressive muscle relaxation (Simon et al., 2022), sleep hypnosis (Besedovsky et al., 2022) and listening to slow wave brain music (Gao et al., 2020), to reduce sleep latency, improve sleep quality, and enhance slow wave sleep (Gao et al., 2020; Besedovsky et al., 2022; Simon et al., 2022). Priming the brain’s sleep network with TUS prior to engaging in these methods might amplify their benefits by creating a brain state more receptive to relaxation with potential benefits such as faster sleep onset and more restorative sleep.

We also envisage that combining offline TUS with Cognitive Behavioural Therapy for Insomnia (CBT-I) might yield greater therapeutic benefits than either intervention alone, even for individuals in the general population who experience suboptimal sleep rather than clinical insomnia (Rossman, 2019; Perrotta and Perri, 2022). In particular, the cognitive restructuring component of CBT-I, which involves changing negative thoughts patterns and behaviours around sleep, might be facilitated by targeting specific regions, such as the nucleus accumbens (NAcc; Yaakub et al., 2024), involved with decision-making and learning which research has shown can be modulated by TUS. Beyond its potential for improving sleep, TUS also hold promise for enhancing self-regulation in other domains, such as emotion regulation, which is intricately linked to sleep in a bidirectional manner (Figure 2).

## 3 Emotion

Tightly linked to healthy sleep patterns, emotions are self-regulated responses that play a crucial role in shaping behaviour. These modulate decision-making (Mitchell, 2011; Arabadzhiyska et al., 2022), direct attention (Mitchell, 2023), facilitate social interactions (Lopes et al., 2011), and enhance episodic memory (Dolcos et al., 2017). Processing of emotions occurs at both conscious (explicit) and unconscious (implicit; Braunstein et al., 2017) levels, and influences the occurrence, duration, intensity, and expression of emotions, to alter or control an emotional response to achieve goal-directed behaviour (Ochsner and Gross, 2005; Gross and Thompson, 2007; Aldao and Plate, 2018).

Emotion regulation involves physiological, cognitive and behavioural processes (Gross and Thompson, 2007), and is recognised as a critical transdiagnostic factor across psychological disorders, including mood, anxiety, substance use, personality, and eating disorders (Aldao et al., 2010). Strengthening the self-regulation of intrinsic emotion can therefore directly enhance quality of life and psychological well-being, by influencing emotional responses so that they are experienced and expressed in a positive manner for healthy functioning (DeSteno et al., 2013). Emotion regulation relies on interacting large-scale neural networks (Morawetz et al., 2020), which can potentially be modulated using NIBS to improve regulatory capacity (Figure 1).

### 3.1 NIBS methods for emotion regulation

TMS and tDCS can target superficial regions of the dorsal surface of the lateral prefrontal cortex (PFC), a region integral to emotional regulation (Phan et al., 2002; Coan and Allen, 2004; Ochsner and Gross, 2005; Price and Drevets, 2012). Stimulating the PFC with these techniques can modulate emotional experience and perception (Peña-Gómez et al., 2011; Vergallito et al., 2018; Chick et al., 2020; Palmisano et al., 2021), potentially improving regulatory control, although findings remain inconsistent and underlying mechanisms are not yet fully established (Qiu et al., 2023). Emotional regulation may be enhanced by reducing negative emotional reactivity to stimuli (Zhang et al., 2022), and tDCS has been shown to decrease stress-related emotional reactivity (Smits et al., 2020).

TMS and tDCS research to date lacks consideration of the distinction between explicit and implicit emotion regulation (Qiu et al., 2023), which are supported by different PFC subregions (Kohn et al., 2014; Etkin et al., 2015). This may, in part, reflect the limited spatial resolution of these techniques. Furthermore, there is paucity of stimulation protocols developed based on specific regulation goals, such as reducing versus intensifying an emotion (Morawetz et al., 2017). Similar to sleep regulation, subcortical limbic structures (e.g., amygdala, hippocampus, thalamus) are implicated in emotional regulation, which tDCS and TMS, unlike TUS, are unable to target (Zhu et al., 2019; Šimić et al., 2021; Ling et al., 2024). While deep brain stimulation (DBS) to these regions has demonstrated therapeutic benefit in extreme cases of emotion dysregulation by changing network-level dynamics (Delaloye and Holtzheimer, 2014; Marquez-Franco et al., 2022), TUS may offer a non-invasive avenue to achieve similar network-level effects.

### 3.2 TUS for emotion regulation: existing research

TMS and tDCS research has provided an important foundation for TUS, demonstrating that stimulation of regions such as the right ventrolateral prefrontal cortex (rVLPFC), associated with negative emotional experience (Wager et al., 2008) and symptoms of depression (Drevets et al., 2008), can reduce negative feelings and negative emotional reactions (Riva et al., 2015; Vergallito et al., 2018). Building on this work, TUS offers enhanced spatial precision, enabling modulation of smaller, functionally specific cortical subregions, such as the right inferior frontal gyrus (rIFG) of the rVLPFC, a central hub for inhibition and cognitive control (Aron et al., 2014). By promoting inhibitory control over emotional processing (Chiu et al., 2008), TUS effectively modulates emotion regulation networks, increasing feelings of happiness and calmness (Sanguinetti et al., 2020).

TUS of subcortical limbic regions central to processing emotional regulation (Šimić et al., 2021), may leverage the advantages of TUS more effectively. Evidence suggests that targeting the amygdala with TUS has the capacity to alter amygdala and broader network activity and connectivity (Kuhn et al., 2023), change emotional reactivity (Hoang-Dang et al., 2024), and decrease levels of anxiety (Chou et al., 2024). Combining TUS with existing psychotherapies and practices could represent a pivotal step towards advancing this field of research, particularly in healthy individuals where the goal is to strengthen self-regulation.

### 3.3 Enhancing emotion self-regulation with TUS

Psychotherapeutic approaches designed to strengthen emotional self-regulation, such as structured psychoeducation and skills-based interventions, may benefit from integration with TUS. These include mindfulness-based cognitive therapy (Sipe and Eisendrath, 2012; Leung et al., 2014) and affect-focused therapies (Iwakabe et al., 2023). Applying TUS to modulate emotion regulation circuits prior to therapy sessions (offline TUS) could improve treatment efficacy by inducing neuroplasticity within relevant neural circuits (Folloni et al., 2019; Fouragnan et al., 2019; Clennell et al., 2021; Yaakub et al., 2023b). Although this has so far been explored primarily in the context of mindfulness-based interventions, extending this approach to other psychotherapeutic modalities warrants further investigation.

TUS has the potential to enhance meditation-based interventions, including mindfulness-based cognitive therapy (Abellaneda-Pérez et al., 2024), which promotes improved emotional regulation (Leung et al., 2014), by modulating the neural circuits underlying attention and emotional control. Meditation is a skill that requires consistent and prolonged practice over many years, with the mindful state deepening over time (Hauswald et al., 2015; Schutte et al., 2020). However, many individuals struggle to initiate or maintain regular practice (Lomas et al., 2015), and some may experience adverse effects depending on the technique used (Palmer et al., 2024). By facilitating mindful states, TUS could make the psychological benefits of meditation more accessible to a wider population (Hauswald et al., 2015; Schutte et al., 2020). Looking ahead, at-home TUS systems integrated with mobile applications may provide cost-effective personalised support for meditation and psychotherapy, particularly when combined with multimodal real-time feedback (e.g., neural activity) to guide self-regulation.

## 4 Discussion

Sleep and emotion are tightly interconnected systems that share overlapping neurochemical and neural substrates. Emerging neurotechnologies such as TUS offer new ways to modulate these processes through enhanced self-regulation (Figure 2). As a promising NIBS technique, TUS can precisely target deep brain structures, distinguishing it from other methods (Figure 1), and induce neuroplasticity that may be further strengthened through combination with neurofeedback and cognitive training. Given their bidirectional relationship, where sleep influences emotional stability and emotion regulation affects sleep quality (Vandekerckhove and Wang, 2017), these intertwined domains present an opportunity for integrated interventions that promote mental resilience and overall well-being.

While the application of TUS in healthy populations remains theoretical, evidence from clinical cohorts with sleep and emotion dysregulation provides a foundation for exploring how this technique could eventually be used to strengthen self-regulatory capacity beyond patient groups. Such preventive applications will depend on continued advances in safety, accessibility, and delivery. Although current protocols require MRI-based targeting and computational modelling, these tools are becoming more widely available and affordable, while rapid advances in transducer design continue to expand feasibility for broader research and clinical implementation.

At the neural level, sleep and emotion regulation rely on interconnected networks involving top-down control from the PFC and subcortical regions such as the amygdala, hypothalamus and thalamus (Goldstein and Walker, 2014; Palmer and Alfano, 2017). Dysregulated sleep can lead to altered connectivity patterns in the PFC and amygdala (Yoo et al., 2007; Motomura et al., 2013), contributing to impairments in related cognitive functions including decision-making, impulsivity (Harrison and Horne, 2000; Killgore et al., 2008) and attentional control (Yoo et al., 2007). Targeting these interconnected networks with TUS could enable precise modulation of limbic–cortical networks, enhancing multiple self-regulatory processes simultaneously. This integrated approach may offer synergistic benefits across emotional balance, cognitive flexibility, and overall well-being.

### 4.1 Future directions

Advancing TUS as a precision neurotechnology will depend on a deeper understanding of the neurobiology of self-regulation and the interindividual variability that shapes neuromodulatory responses. Recent work identifying connectivity-defined biotypes in depression, characterised by distinct frontostriatal and limbic network patterns that predict differential response to TMS, illustrates the potential for brain network profiling to guide stimulation targeting (Drysdale et al., 2017). Whole-brain connectivity measures obtained from task-evoked and resting-state fMRI can serve as individual “fingerprints,” enabling person-specific circuit metrics such as regional dysfunction scores (Finn et al., 2015; Ramduny and Kelly, 2024). Importantly, connectivity between stimulation targets and subcortical hubs like the subgenual cingulate has been shown to prospectively predict antidepressant response to rTMS (Cash et al., 2019), suggesting that similar principles could be applied to refine TUS target selection and parameterisation.

Building on these precedents, connectome-based predictive modelling (Shen et al., 2017; Wang et al., 2021) and machine-learning approaches (Bzdok and Meyer-Lindenberg, 2018) can capture high-dimensional connectivity features that define biotypes and predict neuromodulatory outcomes. Machine learning can advance this approach by efficiently processing large-scale neuroimaging datasets and identifying subtle, high-dimensional patterns of connectivity that define biotypes. Deep learning algorithms, particularly those leveraging convolutional and graph neural networks, could enhance feature extraction from fMRI data, enabling more precise biotype classification and prediction of individual responses to TUS (Drysdale et al., 2017). Integrating multimodal neuroimaging, genetics, and behavioural and cognitive phenotyping through big data frameworks could also reveal previously unrecognised neurobiological subtypes and their regulatory mechanisms. By training predictive models on vast repositories of neuroimaging and clinical datasets, machine learning could refine treatment personalisation, dynamically adapting stimulation parameters to individual brain network configurations (Woo et al., 2017). Ultimately, these computational advancements could drive more effective, data-driven interventions, improving both the precision and efficacy of TUS.

In parallel, continued technical innovation is needed to refine TUS device deign, improve transcranial coupling, and deepen our understanding of underlying brain mechanisms. Determining optimal parameters for excitation and inhibition, and characterising regional and cell-type specific responses (Murphy and de Lecea 2024) are critical next steps. Progress has been constrained by the complexity of the vast parameter space influencing neuromodulatory effects, including intensity, pulse duration, pulse repetition frequency, duty cycle, and carrier frequency (Yoon et al., 2019; Zhang et al., 2021). To accurately neuromodulate subregional areas, advances in skull refraction and attenuation modelling are required to account for individual skull characteristics and resulting transmission losses (Angla et al., 2023; Sigona and Caskey, 2024). Ultimately, the development of at home, image guided TUS systems may enable safe and precise delivery of stimulation, facilitating broader research and preventive applications in healthy individuals.

## 5 Conclusion

Together, recent advances in precision mapping, computational modelling, and device innovation are paving the way for more personalised and scalable applications of TUS. As safety and targeting methods continue to improve, the translation from research to real-world use becomes increasingly feasible. Combining non-invasive TUS with complementary approaches could enable home-based interventions to enhance self-regulation, modulate neural circuits underlying sleep and emotion, and strengthen resilience. These strategies hold promise not only for preventive mental health applications but also for promoting overall well-being across the general population. Continued research and technological innovation will be crucial to optimise safety, precision, and accessibility. With rapid progress in neurotechnology, TUS hold strong potential to deliver practical, data-driven interventions that support healthy brain function and psychological stability in everyday life.

## Data Availability

The original contributions presented in the study are included in the article/supplementary material, further inquiries can be directed to the corresponding author.
